# Getting critical care patients home for end-of-life care

**DOI:** 10.1186/cc12471

**Published:** 2013-03-19

**Authors:** LS Nielsen

**Affiliations:** 1 Kolding Sygehus, Kolding, Denmark

## Introduction

Despite our efforts in making patients healthy and going home, critical illness has a mortality, in Danish ICUs, of 10 to 12%. Approximately 90% of deaths in ICUs happen after life-sustaining therapy has been withheld or withdrawn. Although trying to provide patients and family with what we suppose is a good death, most patients would prefer dying at home, and sometimes patients and family ask for the possibility of doing that. The last 2 years we transferred seven patients from our unit to end-of-life care in their own home.

## Methods

After making the decision of withholding or withdrawing intensive care therapy, the care of the patient changes from an active, medical, technological treatment to relief and care. In that period we determine whether the patient's condition is stable enough to go home. We try to find out if it is a wish for the patient to go home, and if the family has resources to take care of the patient at home. If that is the case, we start planning care at home, arrange for transportation, and contact the primary care physician and nurse. Due to the patient's condition on the days of transferring, we plan following the patient by either a nurse or a doctor.

## Results

Seven terminally ill patients wishing to go home for dying were transferred home. Diagnoses varied: end-stage lung disease, cancer, surgical complications. Ages ranged from 68 to 84 years. All patients survived transport home, and time at home varied from a few hours to 4 days. Later contacts with patients' families indicated that both patient and family were grateful, and that they did not experience the patient having pain or dyspnea at home.

## Conclusion

Sending critically ill patients home to die is not common. Anyhow, our experiences doing that are only positive. Terminally ill patients, awake to make a decision of their own, and in a condition making it possible, should have the choice to go home to die, with our help in logistics, planning and transportation.
